# Efficacy and safety of novel oral anticoagulants in patients with atrial nonvalvular atrial fibrillation and diabetes mellitus: a systematic review and meta-analysis

**DOI:** 10.1186/s12967-022-03652-9

**Published:** 2022-09-30

**Authors:** Xuedong Jia, Zhao Yin, Wan Zhang, Shuzhang Du, Jian Kang

**Affiliations:** Department of Pharmacy, The First Affiliated Hospital of Zhengzhou University, 43 North University Road, Zhengzhou City, 450052 Henan Province China

**Keywords:** New direct oral anticoagulants (NOACs), Warfarin, Atrial fibrillation, Diabetes, Efficacy, Safety, Systematic review, Meta-analysis

## Abstract

**Objective:**

This study incorporates the results of subgroup analyses of currently published randomized controlled trials (RCTs) and real-world cohort studies to compare the effectiveness and safety of new direct oral anticoagulants (NOACs) and warfarin among nonvalvular atrial fibrillation patients with diabetes.

**Methods:**

The PubMed, Embase, Cochrane Library, Web of Science and ClinicalTrials.gov databases were searched. Five retrospective cohort studies and four subgroup analyses of RCTs were included in this meta-analysis.

**Results:**

A meta-analysis of the data of 26,7272 patients showed that for patients with nonvalvular atrial fibrillation and diabetes, NOACs can significantly reduce the incidence of stroke/systemic embolism (SSE), ischaemic stroke, and haemorrhagic stroke compared with warfarin, with no significant difference in major bleeding and all-cause mortality. Additionally, NOACs were superior to warfarin in the incidence of intracranial bleeding, gastrointestinal bleeding, myocardial infarction, and vascular death.

**Conclusions:**

Among nonvalvular atrial fibrillation patients with diabetes, NOACs were associated with a lower risk of SSE versus warfarin, with no significant difference in major bleeding. Therefore, NOACs may be a better clinical choice.

**Supplementary Information:**

The online version contains supplementary material available at 10.1186/s12967-022-03652-9.

## Background

Diabetes increases the risk of atrial fibrillation (AF) and is associated with increased symptom burden, lower quality of life, and increased hospitalization and mortality rates [1]. Among AF patients with diabetes, both thromboembolic and haemorrhagic events were significantly increased [2], indicating that diabetes is an important factor in CHA2DS_2_-VASc bleeding risk scores that are commonly used in patients with AF [3]. Four large phase III clinical trials (Dabigatran etexilate RE-LY [4], Rivaroxaban ROCKET AF [5], Apixaban ARISTOTLE [6], and Edoxaban ENGAGE AF- TIMI 48 [7]) have compared the effectiveness and safety of new direct oral anticoagulants (NOACs) with warfarin, and the results showed noninferiority for safety and efficacy [8]. A previous meta-analysis of the four NOAC trials also found no significant interaction between treatment and diabetes status for stroke/systemic embolism (SSE) or major bleeding [9, 10]. Therefore, current international guidelines recommend the use of NOACs as effective, safer and more convenient alternatives to warfarin among patients with NVAF with diabetes [11, 12].

However, in post hoc analyses of the RE-LY study [15] and ARISTOTLE study [18], diabetes and treatment had a significant interaction for the risk of major bleeding, and the risk of major bleeding for dabigatran 110 mg or apixaban over warfarin was diminished in AF patients comorbid with diabetes. This difference may be attributed to differences in data at baseline (patient age, NOAC dose, patient's underlying cardiovascular disease, the specific definition of major bleeding in the trial, the mean CHADS2 score and the varying degrees of renal metabolism) in diabetic and nondiabetic patients.

Data from real-world studies have shown that NOACs are more effective than warfarin in terms of treatment. However, results regarding the risk of major bleeding have varied widely [13, 16, 17, 19, 20]: some studies have shown that NOACs are better than warfarin in terms of the risk of major bleeding [16, 19, 20], while other studies have reported no difference [13, 17]. To further explore the effectiveness and safety of NOACs in the treatment of patients with NVAF and diabetes, we conducted this systematic review and meta-analysis based on the results of subgroup analyses of randomized controlled trials (RCTs) and real-world data.

## Methods

We conducted a systematic review and meta-analysis in accordance with the Meta-analyses of Observational Studies in Epidemiology guidelines [22]. Our study is registered with PROSPERO (URL: https://www.crd.york.ac.uk/prospero/. Unique identifier: CRD42020192098). Note: When registering for the study, we originally planned to use odds ratio (OR) as an outcome indicator. However, most of the studies we included used hazard ratio (HR) as an outcome indicator. Therefore, we used HR as an outcome indicator to conduct meta-analysis and report the results.

### Literature search strategy

We searched the PubMed, Embase, Cochrane Library, and Web of Science databases from inception through June 2020. The ClinicalTrials.gov databases was also searched for ongoing and unpublished studies. No language restriction was applied. The reference lists of the related studies, reviews and meta-analyses were also examined. The search terms were [“apixaban” or “dabigatran” or “rivaroxaban” or “edoxaban” or “new oral anticoagulants” or “direct oral anticoagulants” or “DOACs” “non-vitamin K oral anticoagulants” or “NOACs”] AND [“Atrial Fibrillation” or “Auricular Fibrillation”] AND [“diabetes mellitus” or “diabetes” or “hyperglycemia”].

### Inclusion and exclusion criteria

The inclusion criteria for the present meta-analysis were as follows: (1) prospective or retrospective cohort studies that started with the recruitment of patients with NVAF in the setting of diabetes who received either NOACs or dose-adjusted warfarin; (2) subgroup analyses of RCTs that compared the risk of efficacy and safety of any NOACs with dose-adjusted warfarin by diabetes status; and (3) investigated NOACs include apixaban, dabigatran, edoxaban, and rivaroxaban. The exclusion criteria were as follows: (1) studies that lack corresponding outcome indicators; (2) duplicate results from the same population; and (3) studies without relevant data after contacting the original author.

### Outcomes

The primary efficacy outcome was the SSE composite measure, and the safety outcome was major bleeding. The secondary efficacy outcomes included ischaemic stroke and haemorrhagic stroke, and the secondary safety outcomes included intracranial bleeding, gastrointestinal bleeding, myocardial infarction and all-cause mortality.

### Data extraction

Data relevant to this study were independently extracted from the screened literature by two reviewers (JXD and YZ) using a data collection sheet in accordance with the recommendations from the Cochrane handbook for systematic reviews of interventions. The following data were extracted: study design, publication year, number of test and control groups, age of test subjects and dose of test drugs, CHADS2 score, baseline characteristics of participants, methods used to identify and verify the diagnosis of NVAF and diabetes. Disagreements were resolved by referring back to the original articles and consensus with a third member of our team (ZW).

### Risk of bias

The inclusion and data extraction of all studies were performed independently by two researchers (JXD and YZ) according to the corresponding criteria, and the original literature or data were checked by a third researcher (ZW) for inconsistencies. The study of subgroup analyses of RCTs was the same as a cross-sectional study, and we assessed the quality of these analyses using the Agency for Health care Research and Quality (AHRQ) quality indicators [23]. The quality of cohort studies was assessed using the Newcastle‒Ottawa Scale (NOS) [24]. Both tools were applied independently by two review authors (JXD and YZ). If the raters disagreed, a third review author (ZW) was consulted.

### Data synthesis and statistical analysis

Meta-analysis was performed using Stata 15.1 SE software (StataCorp, 2017). The generic inverse variance method was used, and hazard ratios (HRs) and 95% confidence intervals (CIs) were used to describe the outcomes. Cochran’s Q test was used for statistical heterogeneity. A value of I^2^ < 50% and P > 0.10 represents low heterogeneity, and in such cases, a fixed effects model was used for meta-analysis; in cases of high heterogeneity, the random effects model was used for meta-analysis. Additionally, subgroup analyses were conducted for both the primary efficacy and safety outcome to explore the heterogeneity among treatment effects. This was carried out based on the possible sources of heterogeneity, including drug type, drug dose and complicating disease of the patient.

## Results

### Literature search

**Fig.1:**
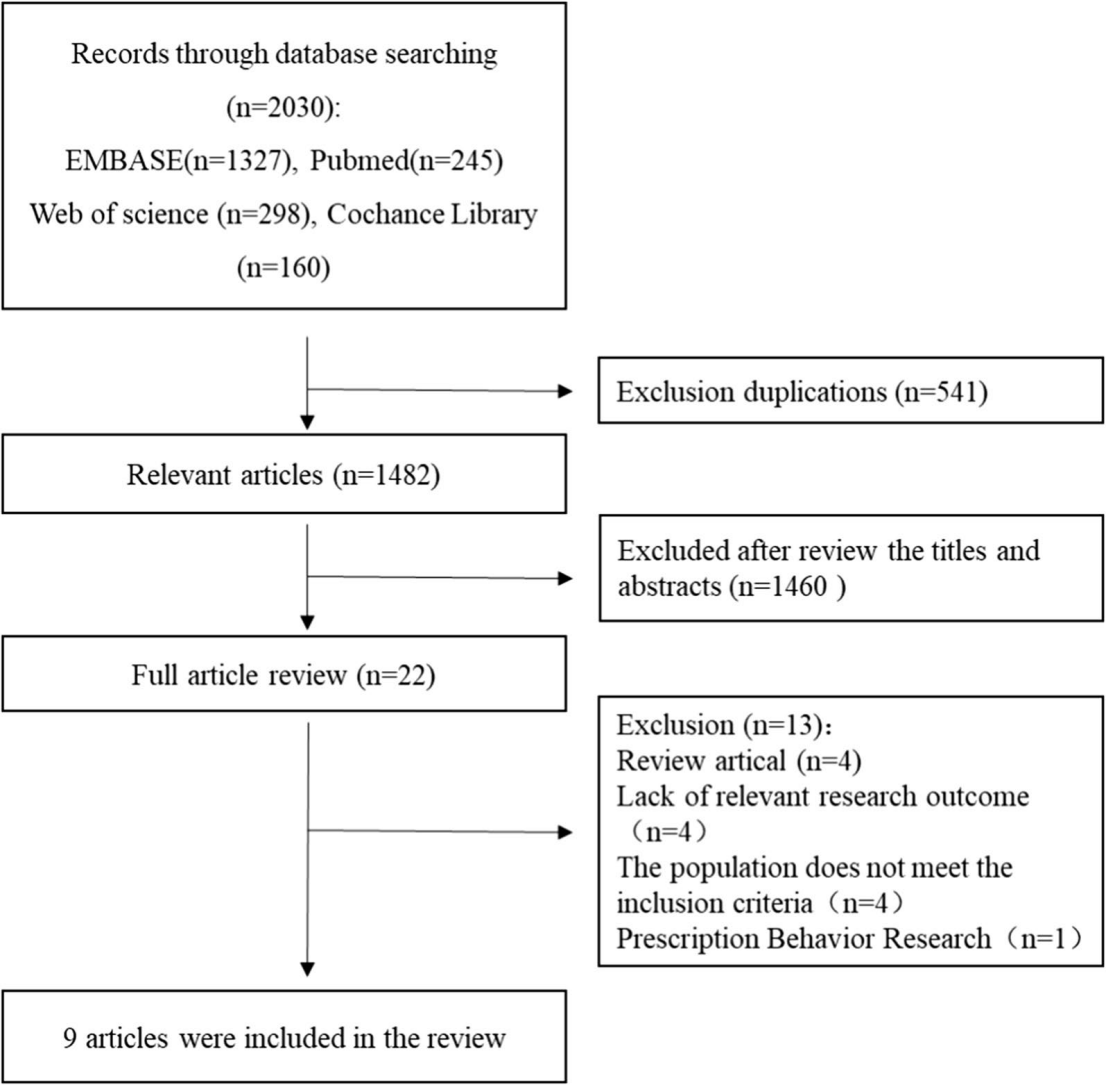
Flow-chart of literature review process

The systematic search yielded 2030 potentially relevant articles; 541 duplicate articles and 1682 other articles were excluded after reading the title and abstract because they clearly did not fulfil the eligibility criteria. A total of 18 articles were retrieved for full-length article review, and 11 articles were excluded at this stage. Ultimately, 5 retrospective cohort studies [13, 16, 17, 19, 20] and 4 subgroup analyses of RCTs [14, 15, 18, 21] were included in the meta-analysis. The literature retrieval, review, and selection process are shown in Fig. 1. The characteristics of the included studies are described in Table 1.

**Table 1:** Baseline characteristics of studies included in the analysis

Study	Type of study	Country	Type of diabetes	Experimental group	Control group	Baseline characteristics of subjects (NOACs VS warfarin)					Follow-up(Year)^c^
				NOACs^a^	Warfarin	Age^b^	Men	CKD	CAD	PAD	
Baker et al. [13]	Retrospective cohorts	US	Type 2 diabetes	Rivaroxaban (n = 10,700, 24.1% received low-dose)	13,946	70 (62, 79) VS 70 (62, 79)	64.7% VS 62.7%	14.0% VS 14.4%	5.3% VS 5.1%	10.7% VS 11.2%	1.4 (0.6, 2.7)
Bansilal et al. [14]	Subgroup of RCT	International multicenter	Type 1 and 2 diabetes	Rivaroxaban (n = 2878, 16% received low-dose)	2817	71 (64, 77) VS 71 (64, 77)	60.8% VS 60.5%	NR	NR	NR	1.9
Brambatti et al. [15]	Subgroup of RCT	International multicenter	Type 1 and 2 diabetes	Dabigatran 110 mg twice daily (n = 1409) Dabigatran 150 mg twice daily (n = 1402)	1410	70.9 ± 8.0 VS 70.9 ± 8.0	NR	NR	NR	NR	2
Chan et al. [16]	Retrospective cohort study	China	Type 1 and 2 diabetes	Apixaban (n = 3249, 66% received low-dose) Dabigatran (n = 6531, 89% received low-dose) Edoxaban (n = 1389, 68% received low-dose) Rivaroxaban (n = 9798, 95% received low-dose)	5812	74.6 ± 10.1 VS 74.5 ± 10.3	53.7% VS 53.5%	22.7% VS 23.6%	13.9%VS 14.5%	9.6% VS 9.4%	NR
Coleman et al. [17]	Retrospective cohort study	US	Type 1 and 2 diabetes	Rivaroxaban (n = 5517, 20% received low-dose)	5517	70 (62, 78) VS 70 (62, 78)	63.3% VS 63.5%	16.2% VS 16.0%	NR	20.3% VS 21.1%	1.5 (0.7, 2.7)
Ezekowitz et al. [18]	Subgroup of RCT	International multicenter	Type 1 and 2 diabetes	Apixaban (n = 2559, NR percentage of low-dose)	2263	69 (63, 75) VS 69 (62, 75)	64.4% VS 65.7%	53.2% VS 51.3%	38.3% VS 39.5%	7.1% VS 7.4%	1
Hsu et al. [19]	Retrospective cohort study	China	Type 2 diabetes	Dabigatran (n = 305, 88.5% received low-dose)	305	75.1 ± 9.1 VS 73.9 ± 8.7	56.4% VS 49.2%	38.4% VS 38.7%	64.3% VS 62.3%	72.1% VS 72.5%	NR
				Rivaroxaban (n = 300, 87.5%received low-dose)	301	75.2 ± 8.7 VS 74.4 ± 8.2	44.7% VS 52.5%	43.3% VS 40.9%	68.3% VS 65.8%	67.7% VS 69.1%	
Lip et al. [20]	Retrospective cohort study	US	Type 1 and 2 diabetes	Apixaban (n = 35,269, 25.2% received low-dose)	35,269	75.8 ± 9.0 VS 75.8 ± 8.9	53.8% VS 53.7%	35.6% VS 35.8%	57.0% VS 56.4%	26.6% VS 27.5%	0.5 ± 0.2 VS 0.7 ± 0.6
				Dabigatran (n = 12,954, 19% received low-dose)	12,954	73.7 ± 9.1 VS 73.9 ± 9.3	57.6% VS 57.8%	25.8% VS 26.0%	51.8% VS 51.2%	22.7% VS 23.8%	0.6 ± 0.6 VS 0.7 ± 0.6
				Rivaroxaban (n = 44,412, 32% received low-dose)	44,412	75.2 ± 8.9 VS 75.3 ± 8.9	55.2% VS 55.1%	31.2% VS 30.9%	54.4%VS 54.4%	26.0% VS 26.1%	0.6 ± 0.6 VS 0.7 ± 0.6
Plitt et al. [21]	Subgroup of RCT	International multicenter	Type 1 and 2 diabetes	Edoxaban (n = 2559, NR percentage of low-dose)	2521	70 (63–76) VS 70 (63–76)	62.6% VS 65.1%	15.8% VS 14.1%	NR	NR	NR

*CKD* Chronic kidney disease; *CAD* Coronary artery disease; *PAD* Peripheral artery disease; *NOACs* non-vitamin K oral anticoagulants, *NR* Not reported

^a^Standard dose: 5 mg apixaban twice daily, 150 mg dabigatran twice daily, 20 mg rivaroxaban once-daily, 60 mg edoxaban once-daily. Lower dose: 2.5 mg apixaban twice daily, 75 mg dabigatran twice daily, 10 or 15 mg rivaroxaban once-daily, 30 mg edoxaban once-daily

^b^Data are presented as mean ± SD or the median (25%, 75% range)

^c^The presentation of data varies by primary literature

### Document quality assessment

The quality of the included subgroup of RCTs was assessed using the AHRQ tool, and cohort studies were independently assessed according to the NOS. The results of their quality assessment are shown in Additional file 1: Tables S1 and S2.

### Primary outcomes

#### Stroke/systemic embolism (SSE)

**Fig. 2:**
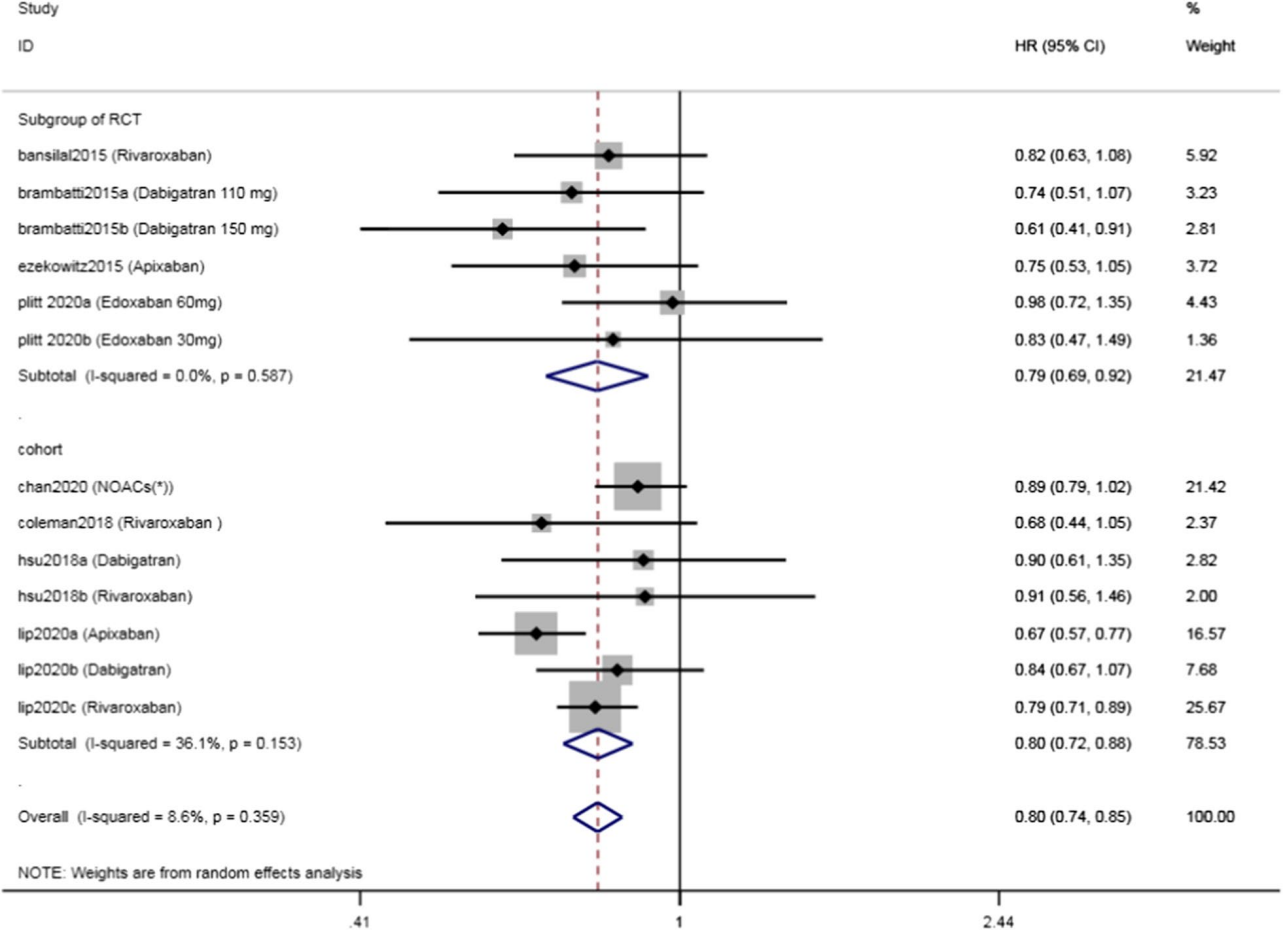
Forest plot of the risk of stroke/systemic embolism among NVAF patients with diabetes on DOACs versus warfarin

A total of 13 subgroups from 8 studies [14–21] that contained 24 3837 participants were included in this analysis. The heterogeneity was low (I^2^ = 8.6%, p = 0.359), so we used the fixed effects model to assess the outcome. NOACs significantly reduced the risk of SSE when compared with warfarin among patients with NVAF and diabetes (pooled HR = 0.80, 95% CI 0.74, 0.85). Among the included studies, there were 4 subgroups of RCTs and 4 cohort studies, and the I^2^ was 0% and 36.1% for the subgroup of RCTs and cohort studies, respectively, suggesting significant heterogeneity among the cohort studies. However, the combined analysis results of the two types of studies were consistent (pooled HR = 0.79, 95% CI 0.69, 0.92 for RCTs; [pooled HR = 0.80, 95% CI 0.72, 0.88 for cohort studies) (Fig. 2).

Subgroup analysis was performed to determine whether different NOACs or different drug doses were superior to warfarin for SSE. Subgroup analysis results based on drug dose show that both standard-dose and lower-dose (reduced dose by renal function or patient age or patient weight or concomitant use of strong P-gp inhibitor) NOACs can reduce the risk of SSE compared with warfarin, consistent with the overall results. Subgroup analysis results based on the type of drugs showed that rivaroxaban, dabigatran and apixaban significantly reduced the risk of SSE, consistent with the overall results when compared with warfarin, while edoxaban showed a different effect from warfarin, with a pooled HR = 0.87 95% CI 0.69, 1.10) (Additional file 1: Table S3).

#### Major bleeding

**Fig. 3:**
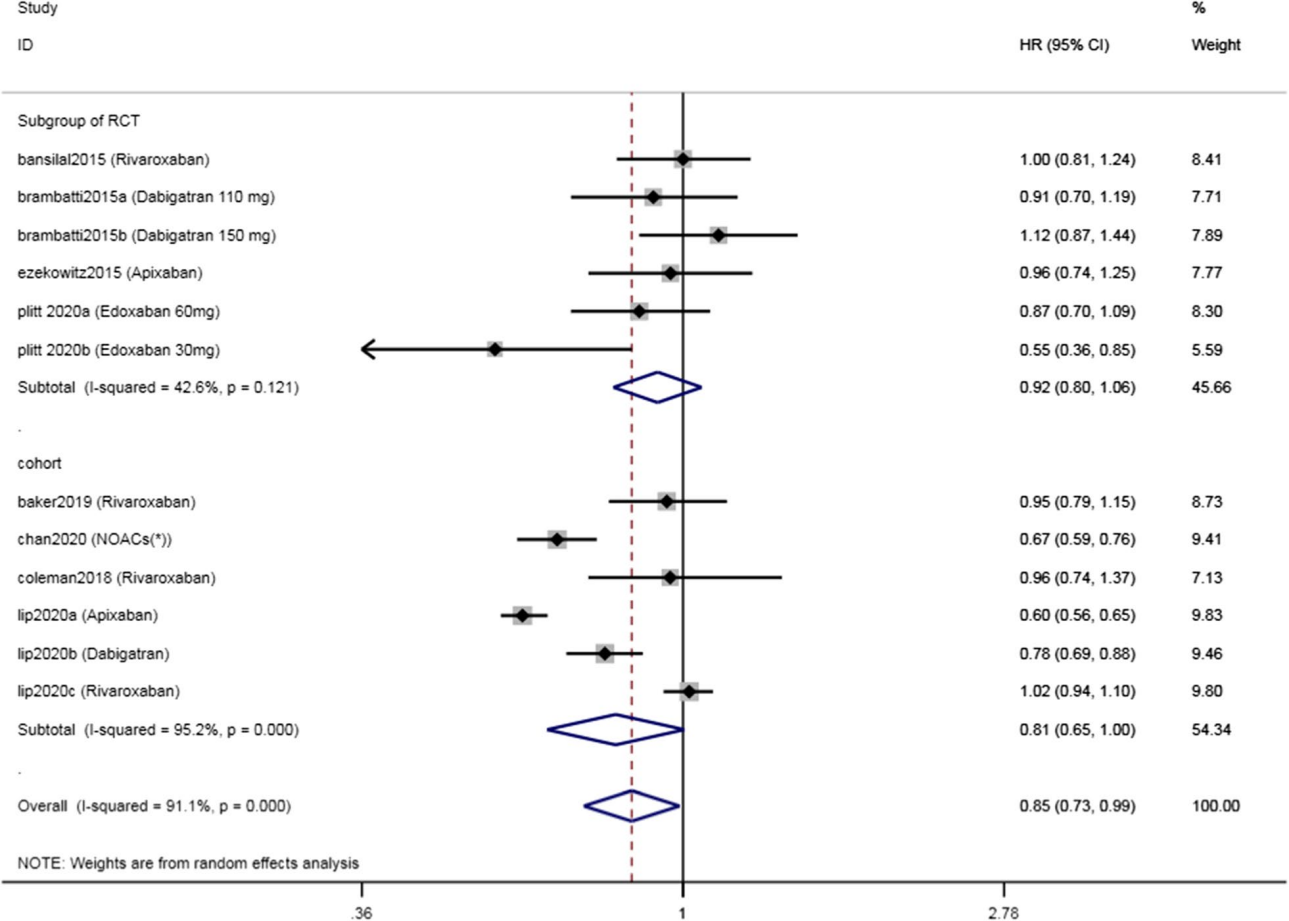
Forest plot of the risk of major bleeding among NVAF patients with diabetes on NOACs versus warfarin

A total of 12 subgroups from 8 studies [13–18, 20, 21] that contained 26,7272 participants were included in this analysis. The heterogeneity was high (I^2^ = 91.1%, p = 0.000), so we used the random effects model to assess the outcome. NOACs can reduce the risk of major bleeding when compared with warfarin among patients with NVAF and diabetes (pooled HR = 0.85, 95% CI 0.73, 0.99). Among the included studies, there were 4 subgroups of RCTs and 4 cohort studies, and the I^2^ was 42.6% and 95.2% for the subgroup of RCTs and cohort studies, respectively, suggesting significant heterogeneity among the studies. When performing subgroup analysis by study type, the risk of major bleeding was not different between NOACs and warfarin (pooled HR = 0.92, 95% CI 0.80, 1.06 for RCTs; pooled HR = 0.81, 95% CI 0.65, 1.00 for cohort studies) (Fig. 3).

Subgroup analysis was performed to determine whether different types of NOACs, different drug doses or complicating disease can influence the risk of major bleeding. Subgroup analysis results based on different drug doses showed that the risk of major bleeding was not different between NOACs and warfarin in either the standard-dose or lower-dose subgroups. Subgroup analysis results based on the type of drugs showed that rivaroxaban, dabigatran and edoxaban showed no difference in the risk of major bleeding, consistent with the overall results when compared with warfarin, while apixaban significantly reduced the risk of bleeding (pooled HR = 0.71, 95% CI 0.54, 0.93). The subgroup analysis results based on complicating disease showed that complicating peripheral artery disease (PAD) did not influence major bleeding, and for chronic kidney disease (CKD) patients, NOACs significantly reduced the risk (pooled HR = 0.75, 95% CI 0.61, 0.92) (Additional file 1: Table S4).

### Secondary outcomes

#### Ischaemic stroke

**Fig. 4:**
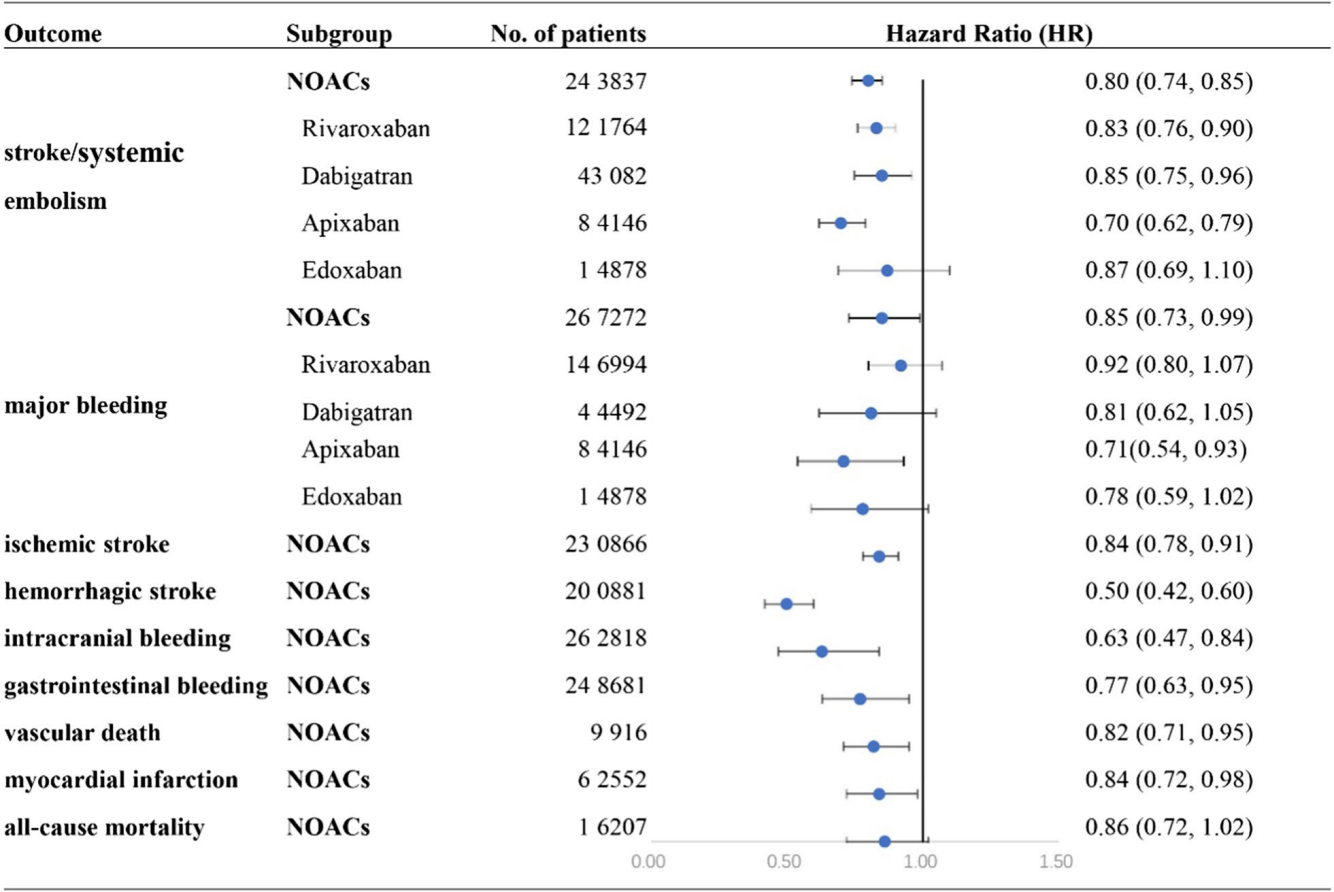
Efficacy and safety of NOACs in patients with NVAF and diabetes mellitus

A total of 8 subgroups from 5 studies [13–15, 17, 20] that contained 23 0866 participants were included in this analysis. The heterogeneity was low (I^2^ = 4.8%, p = 0.393), so we used the fixed effects model to assess the outcome. The risk of ischaemic stroke among patients with NVAF and diabetes who received NOACs was significantly reduced when compared with those who received warfarin (pooled HR = 0.84, 95% CI 0.78, 0.91) (Fig. 4, Additional file 1: Fig. S1).

#### Haemorrhagic stroke

A total of 7 subgroups from 4 studies [14, 15, 17, 20] that contained 200,881 participants were included in this analysis. The heterogeneity was low (I^2^ = 3.4%, p = 0.400), so we used the fixed effects model to assess the outcome. The risk of haemorrhagic stroke among patients with NVAF and diabetes who received NOACs was significantly reduced when compared with those who received warfarin (pooled HR = 0.50, 95% CI 0.42, 0.60) (Fig. 4, Additional file 1: Fig. S2).

#### Intracranial bleeding

A total of 15 subgroups from 9 studies [13–21] that contained 26 2818 participants were included in this analysis. The heterogeneity (I^2^ = 88.5%, p < 0.001) was low, so we used the random effects model to assess the outcome. The risk of intracranial bleeding among patients with NVAF and diabetes who received NOACs was significantly reduced when compared with those who received warfarin (pooled HR = 0.63, 95% CI 0.47, 0.84) (Fig. 4, Additional file 1: Fig. S3).

#### Gastrointestinal bleeding

A total of 10 subgroups from 6 studies [13, 16, 17, 19–21] that contained 24 8681 participants were included in this analysis. The heterogeneity was low (I^2^ = 88.3%, p < 0.001), so we used the random effects model to assess the outcome. The risk of gastrointestinal bleeding among patients with NVAF and diabetes who received NOACs was significantly reduced when compared with those who received warfarin (pooled HR = 0.77, 95% CI 0.63, 0.95) (Fig. 4, Additional file 1: Fig. S4).

#### Vascular death

A total of 3 subgroups from 2 studies [14, 15] that contained 9916 participants were included in this analysis. The heterogeneity was low (I^2^ = 0, p = 0.918), so we used the fixed effects model to assess the outcome. The risk of vascular death among patients with NVAF and diabetes who received NOACs was significantly reduced when compared with those who received warfarin (pooled HR = 0.82, 95% CI 0.71, 0.95) (Fig. 4, Additional file 1: Fig. S5).

#### Myocardial infarction

A total of 6 subgroups from 5 studies [13, 14, 16, 18, 19] that contained 6 2552 participants were included in this analysis. The heterogeneity was low (I^2^ = 0, p = 0.716), so we used the fixed effects model to assess the outcome. The risk of myocardial infarction among patients with NVAF and diabetes who received NOACs was significantly reduced when compared with those who received warfarin (pooled HR = 0.84, 95% CI 0.72, 0.98) (Fig. 4, Additional file 1: Fig. S6).

#### All-cause mortality

A total of 7 subgroups from 4 studies [14, 18, 19, 21], which contained 1 6207 participants, were included in this analysis. The heterogeneity was low (I^2^ = 59.7%, p = 0.021), so we used the random effects model to assess the outcome. The risk of all-cause mortality among patients with NVAF and diabetes who received NOACs was similar to those who received warfarin (pooled HR = 0.86, 95% CI 0.72, 1.02) (Fig. 4, Additional file 1: Fig. S7).

## Discussion

Diabetes is an independent risk factor for stroke in patients with NVAF. Studies have shown that the risk of stroke in patients with NVAF combined with diabetes is increased by approximately 70% [2]. Therefore, prevention of stroke is the key to patients with NVAF and diabetes, anticoagulation therapy is the core measure for the prevention of stroke, and it can significantly reduce the risk of stroke and the mortality of patients with NVAF [25]. Warfarin was the cornerstone of oral anticoagulant therapy before the launch of NOACs. Since rivaroxaban was approved for market use in 2010, NOACs have been developed rapidly. Compared with warfarin, these drugs have many advantages, such as more predictable pharmacodynamics, fewer drug and food interactions and the lack of need for routine laboratory monitoring. Therefore, the 2018 European Heart Rhythm Association Room Fibrillation anticoagulation guidelines refer to the recommendation of NOACs as the first choice for stroke prevention in patients with NVAF [26]. Previous studies have shown that when warfarin is used for patients with NVAF with diabetes, it may be more difficult to achieve anticoagulation standards, and the compliance rate is low, which further increases the risk of anticoagulation failure [27]; thus, NOACs have a superior application advantage in patients with NVAF and diabetes. The results of our study further prove the effectiveness and safety of NOACs and can provide important evidence-based guidance for clinical applications.

In this study, a meta-analysis of the data of 26,7272 patients showed that for patients with NVAF and diabetes, NOACs can significantly reduce the incidence of SSE, ischaemic stroke, and haemorrhagic stroke, intracranial bleeding, gastrointestinal bleeding, myocardial infarction, and vascular death compared to warfarin. However, only apixaban had a lower risk of major bleeding than warfarin. Dabigatran, rivaroxaban and edoxaban had a similar risk of major bleeding to warfarin. The all-cause mortality of NOACs also did not show an advantage compared with warfarin.

A combined analysis of the results of the RE-LY [4], ROCKET AF [5], ARISTOTLE [6], and ENGAGE AF- TIMI 48[7] trials showed that the comprehensive risk of SSE in diabetic patients treated with NOACs was 3.16% (9096 patients received NOCAs) and 3.96% among patients treated with warfarin (8990 patients treated with warfarin) (RR = 0.80; 95% CI 0.69, 0.93) [28], indicating that NOACs have a slight advantage over warfarin. Several previous studies [10, 29, 30] also showed that the use of NOACs and vitamin K antagonists (VKA) in patients with NVAF has a similar risk of SSE and major bleeding in diabetes (RR = 0.97 95% CI 0.79, 1.18)) and nondiabetes (RR = 0.76 95% CI 0.65, 0.88) patients. Our study is consistent with the above results. For patients with NVAF and diabetes, the use of NOACs can reduce the risk of SSE without increasing the incidence of major bleeding.

The meta-analysis results of Ruff et al. [29] showed that the use of NOACs in patients with NVAF can significantly reduce all-cause mortality (RR = 0.90, 95% CI 0.85, 0.95; p = 0.0003) but increased gastrointestinal bleeding (RR = 1.25, 95% CI 1.01, 1.55; p = 0.04). However, the results of our study showed that NOACs did not increase the incidence of gastrointestinal bleeding and had no difference in all-cause mortality compared with warfarin. The cause might be that the previous studies were all RCTs, the inclusion criteria of patients were relatively strict, and in our study, the population included was less restrictive and more representative of the real-world population. A study by Patti et al. [10] showed that NOACs did not have advantages for the occurrence of ischaemic stroke and intracranial bleeding compared to warfarin, while the results of our study showed that the use of NOACs was superior to warfarin in terms of the incidence of ischaemic stroke and intracranial bleeding. This difference may be related to the small sample size of the previous study and the large sample size of our study.

The results of our study show that for patients with CKD, the safety of NOACs is higher than that of warfarin, and the risk of major bleeding is lower. However, the results of the study should be interpreted carefully because NOACs have strict limitations on the renal function of patients. For patients with severe renal insufficiency, the safety and effectiveness of NOACs are relatively lacking, so in real-world studies, patients who take NOACs may have better renal function status than those who take warfarin. The warfarin group may include more patients with end-stage renal disease or renal failure, so the effectiveness and safety of the two types of drugs used by patients with CKD is insufficient. A previous meta-analysis showed that NOACs did not differ from warfarin in reducing SSE (RR = 0.81, 95% CI 0.65, 1.00) or major bleeding (RR = 0.79, 95% CI 0.59, 1.04) [31]. The newly published retrospective study based on a database system [32] included 21,733 patients with NVAF with different CKD levels. The results showed that compared with warfarin, NOAC use in patients with impaired renal function was associated with a lower risk of mortality and major bleeding that required hospitalization in patients with all kidney function levels (eGFR > 60%, eGFR > 30–60% and eGFR ≤ 30% or on dialysis). NOACs seem to show clinical advantages in people with renal insufficiency. However, eGFR ≤ 30% or on dialysis patients accounted for only 7.0% in this study, and there was no analysis of CKD stage 5 patients with eGFR ≤ 15%. There is currently no evidence of the use of NOACs in CKD stage 5 patients with an eGFR ≤ 15%, so it is necessary to strengthen the monitoring of CKD stage 5 patients, and more research evidence is needed to support the effectiveness and safety of NOACs in CKD patients.

Medication compliance is an important factor affecting drug efficacy and safety. The results of the meta-analysis on NOAC medication compliance showed that the overall compliance with NOACs was significantly higher than that with vitamin K antagonists (OR = 1.44; 95% CI 1.12–0.86]. Additionally, NOAC nonadherence was associated with an increased risk of stroke (HR = 1.39; 95% CI 1.06–1.81) [33]. Although NOACs improved compliance compared with warfarin and have certain advantages in clinical application, the current study showed that the overall population was more likely to have high medication compliance, so it is still necessary for medical staff to strengthen the education of medication patients in the future, improve patients’ awareness of compliance, and ensure the effective and safe application of drugs in clinical practice.

Although we performed a systematic search and detailed analysis, this study has some limitations. (1) All the included studies were not RCTs, and there may be an imbalance in the inclusion of subjects. The cohort studies included in the analysis were not prospectively designed, and the research methods were not uniform. Some studies used propensity score matching (PSM) to group subjects, and some used natural grouping, thereby introducing potential bias in the analysis. (2) The study did not analyse the patient’s diabetes type, blood sugar control status, or the impact of the current hypoglycaemic program on the results. These factors may have a great impact on the results. (3) Finally, a study from China [34] was not included in the analysis even though it met our inclusion criteria because the outcome of the study was not reported using HRs; thus, we could not combined the data from that study with the data from the included studies. The exclusion of the study from China may have introduced some study bias. Therefore, we still need to be cautious when interpreting the evidence of this study. More large-scale, multicentre, random, double-blind experiments are needed to provide additional evidence.

## Conclusion

In the present study, we performed a systematic assessment regarding the efficacy and safety of NOACs in patients with NVAF and diabetes mellitus. A total of 5 retrospective cohort studies and 4 subgroup analyses of RCTs were included in this study. The results of our meta-analysis indicated that among NVAF patients with diabetes, NOACs were associated with a lower risk of SSE, apixaban had a lower risk of major bleeding, and dabigatran, rivaroxaban and edoxaban had a similar risk of major bleeding compared with warfarin. This suggests that NOACs may be a better choice for anticoagulation in patients with NVAF and diabetes.

## Supplementary Information

**Additional file 1: Table S1.** Newcastle-Ottawa Scale (NOS) quality scale for cohort studies. **Table S2.** Agency for Healthcare Research and Quality (AHRQ) quality scale for sub-analysis of RCTs. **Table S3.** Stratified analysis of the risk of SSE among NVAF patients with diabetes on NOACs versus warfarin according to drug type and dose. **Table S4.** Stratified analysis according to drug type, drug dose and basic condition. **Figure S1.** Forest plot of the risk of ischemic stroke among NVAF patients with diabetes on NOACs versus warfarin. **Figure S2.** Forest plot of the risk of hemorrhagic stroke among NVAF patients with diabetes on NOACs versus warfarin. **Figure S3.** Forest plot of the risk of intracranial bleeding among NVAF patients with diabetes on NOACs versus warfarin. **Figure S4.** Forest plot of the risk of gastrointestinal bleeding among NVAF patients with diabetes on NOACs versus warfarin. **Figure S5.** Forest plot of the risk of vascular death among NVAF patients with diabetes on NOACs versus warfarin. **Figure S6.** Forest plot of the risk of myocardial infarction among NVAF patients with diabetes on NOACs versus warfarin. **Figure S7.** Forest plot of the risk of all-cause mortality among NVAF patients with Diabetes on NOACs versus warfarin.

## Data Availability

Not applicable.
