# Supplementary material for: Efficacy and safety of novel oral anticoagulants in patients with atrial nonvalvular atrial fibrillation and diabetes mellitus: a systematic review and meta-analysis
Source: J Transl Med. 2022 Sep 30;20:441. doi: 10.1186/s12967-022-03652-9 (PMC9524066; doi:10.1186/s12967-022-03652-9)
Supplement: Supplementary file 1 — Additional file 1: Table S1. Newcastle-Ottawa Scale (NOS) quality scale for cohort studies. Table S2. Agency for Healthcare Research and Quality (AHRQ) quality scale for sub-analysis of RCTs. Table S3. Stratified analysis of the risk of SSE among NVAF patients with diabetes on NOACs versus warfarin according to drug type and dose. Table S4. Stratified analysis according to drug type, drug dose and basic condition. Figure S1. Forest plot of the risk of ischemic stroke among NVAF patients with diabetes on NOACs versus warfarin. Figure S2. Forest plot of the risk of hemorrhagic stroke among NVAF patients with diabetes on NOACs versus warfarin. Figure S3. Forest plot of the risk of intracranial bleeding among NVAF patients with diabetes on NOACs versus warfarin. Figure S4. Forest plot of the risk of gastrointestinal bleeding among NVAF patients with diabetes on NOACs versus warfarin. Figure S5. Forest plot of the risk of vascular death among NVAF patients with diabetes on NOACs versus warfarin. Figure S6. Forest plot of the risk of myocardial infarction among NVAF patients with diabetes on NOACs versus warfarin. Figure S7. Forest plot of the risk of all-cause mortality among NVAF patients with Diabetes on NOACs versus warfarin. [file 12967_2022_3652_MOESM1_ESM.docx]

Table S1 Newcastle-Ottawa Scale (NOS) quality scale for cohort studies

| Study | **Selection of cohort** | | | | **Comparability** | **Outcomes** | | | Score |
| --- | --- | --- | --- | --- | --- | --- | --- | --- | --- |
|  | Representativeness of the exposed | Selection of the non exposed | Ascertainment of exposure | Initially healthy  subjects | Comparability of cohorts | Assessment of outcome | long enough follow-up | Adequacy follow up |  |
| baker2019 | 1 | 1 | 1 | 1 | 2 | 1 | 0 | 0 | 7 |
| chan2020 | 1 | 1 | 1 | 1 | 2 | 1 | 1 | 0 | 8 |
| coleman2018 | 1 | 1 | 1 | 1 | 2 | 1 | 1 | 0 | 8 |
| hsu2018 | 1 | 1 | 1 | 1 | 2 | 1 | 0 | 0 | 7 |
| lip2020 | 1 | 1 | 1 | 1 | 2 | 1 | 1 | 0 | 8 |

Table S2 Agency for Healthcare Research and Quality (AHRQ) quality scale for sub-analysis of RCTs

| **study** | Define the source of information | List inclusion and exclusion criteria for exposed and unexposed subjects or refer to previous publications | Indicate time period used for identifying patients | Indicate whether or not subjects were consecutive if not population-based | Indicate if evaluators of subjective components of study were masked to other aspects of the status of the participants | Describe any assessments undertaken for quality assurance purposes | Explain any patient exclusions from analysis | Describe how confounding was assessed and/or controlled. | If applicable, explain how missing data were handled in the analysis | Summarize patient response rates and completeness of data collection | Clarify what follow-up, if any, was expected and the percentage of patients for which incomplete data or follow-up was obtained |
| --- | --- | --- | --- | --- | --- | --- | --- | --- | --- | --- | --- |
| **bansilal2015** | yes | yes | unclear | yes | yes | no | yes | yes | unclear | yes | yes |
| **brambatti2015** | yes | yes | unclear | yes | yes | no | yes | yes | unclear | yes | yes |
| **ezekowitz2015** | yes | yes | unclear | yes | yes | no | yes | yes | unclear | yes | yes |
| **plitt 2020** | yes | yes | unclear | yes | yes | no | yes | yes | unclear | yes | yes |

Table S3 Stratified analysis of the risk of SSE among NVAF patients with diabetes on NOACs versus warfarin according to drug type and dose

| **Drug Type** | **HR** | **I^2^, P** |
| --- | --- | --- |
| Rivaroxaban | 0.83(0.76, 0.90) | 0.0%, 0.510 |
| Dabigatran | 0.85(0.75, 0.96) | 13.6%, 0.328 |
| Apixaban | 0.70(0.62, 0.79) | 0.0%, 0.622 |
| Edoxaban | 0.87(0.69, 1.10) | 0.0%, 0.476 |
| **Drug Dose** | **HR** | **I^2^, P** |
| Standard-dose | 0.77(0.67, 0.89) | 50.1%, 0.075 |
| Lower-dose | 0.73(0.64, 0.83) | 7.3%, 0.370 |

Table S4 Stratified analysis according to drug type, drug dose and basic condition

| **Drug Type** | **HR** | I^2^, P |
| --- | --- | --- |
| Rivaroxaban | 0.92(0.80, 1.07) | 73.8%, 0.004 |
| Dabigatran | 0.81(0.62, 1.05) | 86.9%, 0.000 |
| Apixaban | 0.71(0.54, 0.93) | 83.0%, 0.003 |
| Edoxaban | 0.78(0.59, 1.02) | 45.6%, 0.159 |
| **Drug Dose** |  |  |
| Standard-dose | 0.88(0.71, 1.10) | 92.2%, 0.000 |
| Lower-dose | 0.79(0.56, 1.06) | 92.4%, 0.000 |
| **Complicating Disease** |  |  |
| PAD | 0.95(0.42, 2.14) | 83.1%, 0.015 |
| No PAD | 0.76(0.58, 1.01) | 79.8%, 0.026 |
| CKD | 0.75(0.61, 0.92) | 0.0%, 0.470 |
| No CKD | 0.80(0.51, 1.27) | 91.4%, 0.001 |

PAD: Complicating Peripheral artery Disease, CKD: Chronic Kidney Disease


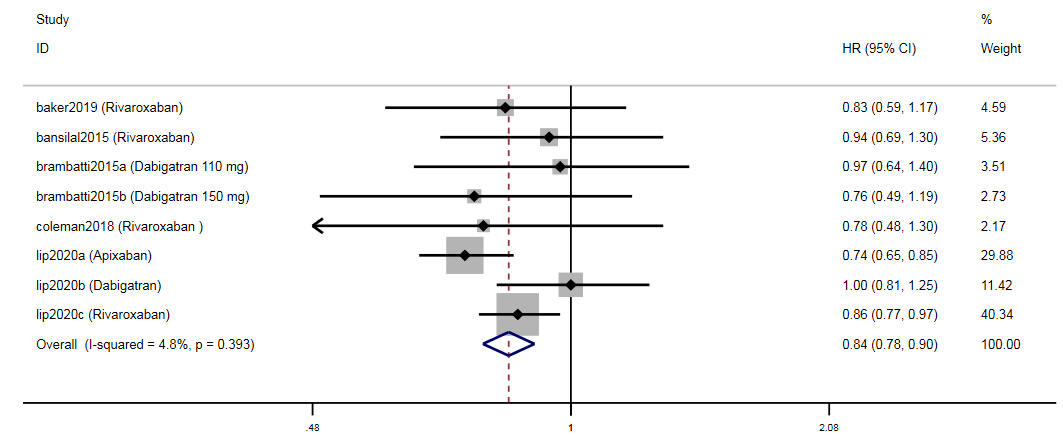


Fig. S1 Forest plot of the risk of ischemic stroke among NVAF patients with diabetes on NOACs versus warfarin.


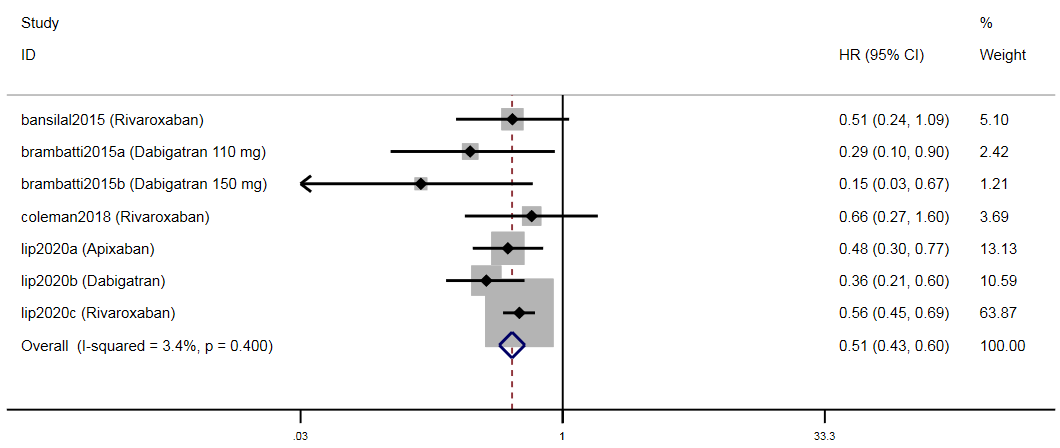


Fig. S2 Forest plot of the risk of hemorrhagic stroke among NVAF patients with diabetes on NOACs versus warfarin.


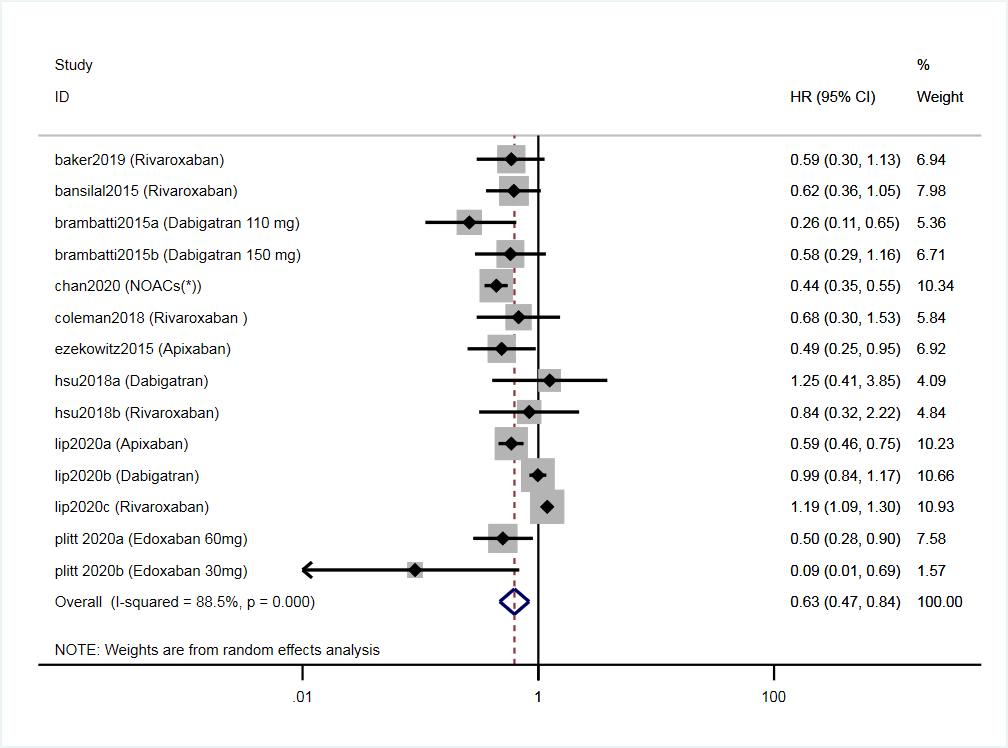


Fig. S3 Forest plot of the risk of intracranial bleeding among NVAF patients with diabetes on NOACs versus warfarin.


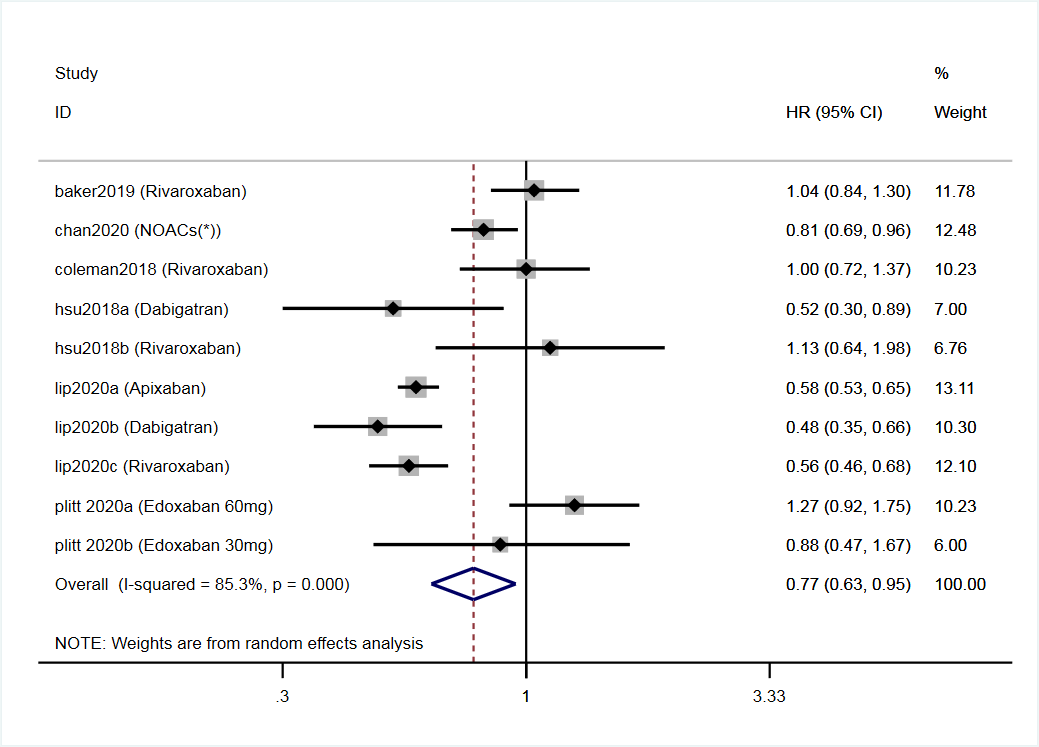


Fig. S4 Forest plot of the risk of gastrointestinal bleeding among NVAF patients with diabetes on NOACs versus warfarin.


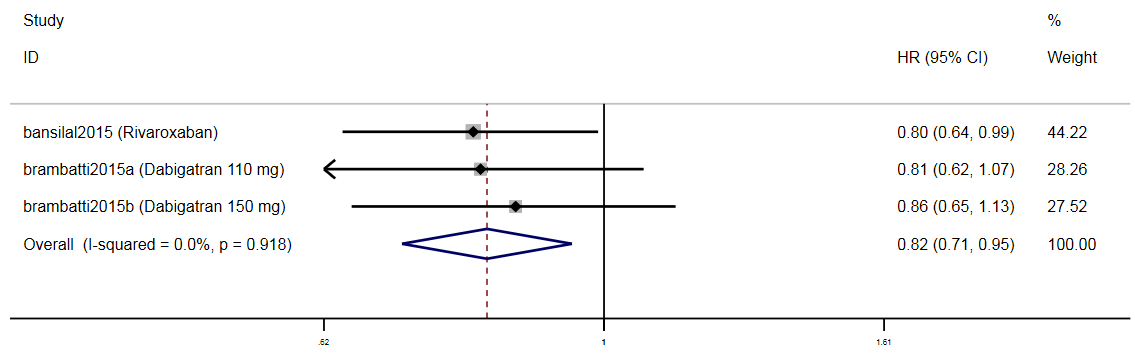


Fig. S5 Forest plot of the risk of vascular death among NVAF patients with diabetes on NOACs versus warfarin.


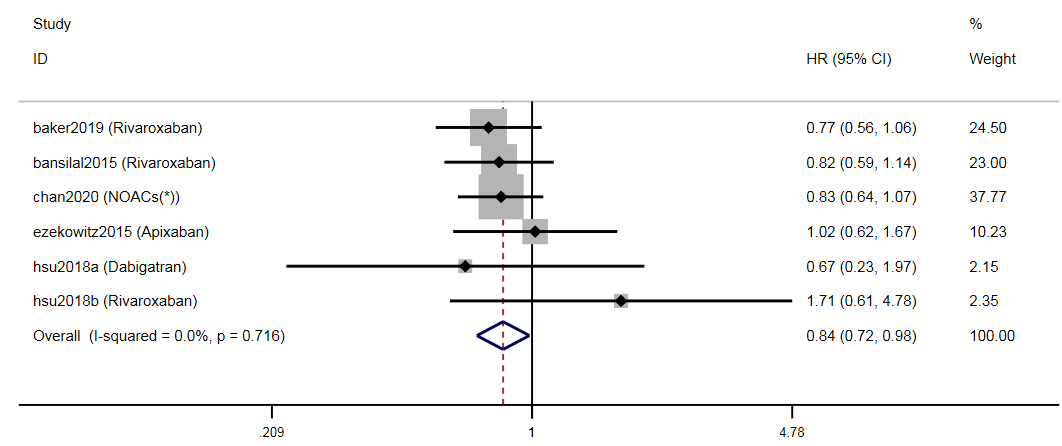


Fig. S6 Forest plot of the risk of myocardial infarction among NVAF patients with diabetes on NOACs versus warfarin.


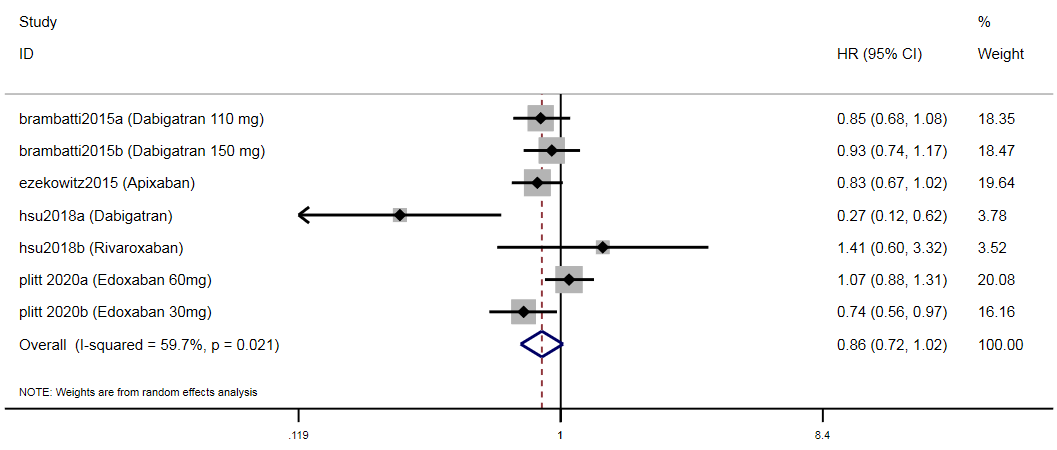


Fig. S7 Forest plot of the risk of all-cause mortality among NVAF patients with Diabetes on NOACs versus warfarin.
